# Gynecological and psycho-sexual aspects of women with history of anorectal malformations

**DOI:** 10.1007/s00383-021-04905-2

**Published:** 2021-04-26

**Authors:** Noemi Bicelli, Emanuele Trovalusci, Monica Zannol, Piergiorgio Gamba, Gianna Bogana, Cinzia Zanatta, Paola Midrio

**Affiliations:** 1 Pediatric Surgery, Cà Foncello Hospital, Treviso, Italy; 2 Obstetrics&Gynecology, Cà Foncello Hospital, Treviso, Italy; 3 Pediatric Surgery, Università Di Padova, Padova, Italy; 4 Obstetrics&Gynecology, Azienda Ospedaliera, Padova, Italy

**Keywords:** Anorectal malformation, Adult life, Gynecological aspects, Sexuality, Psychological well-being

## Abstract

**Purpose:**

Women with anorectal malformation (ARM) are expected to have a normal life span, therefore, gynecological and psycho-sexual issues are also important. Aim of the study was to assess these aspects in adult females with history of ARM.

**Methods:**

Thirty-seven women from two ARM referral centers, aged ≥ 16, were identified. Gynecologic visit, cervicovaginal swab, pelvic ultrasound, FSH, LH, prolactin, progesterone, 17–β–estradiol, DHEAS, testosterone, TSH during follicular and luteal phases, and administration of FSFI questionnaire to screen the female sexual functioning were performed. Data were compared with six controls.

**Results:**

Nineteen patients, mean age 21.7 (16–45), participated to the study. Associated anomalies, mostly affecting limbs, vertebrae and genitalia, were present in 57.8% of cases. Mullerian anomalies were retrieved in 36.8%. Hormones’ levels were normal. Concerning sexual functioning, four women (21%) reported dyspareunia or impossible penetration, four did not answer the FSFI questionnaire due to lack of confidence about their sexuality, and three scored lower than the cut-off value for female sexual function.

**Conclusion:**

This study confirms the importance of a multidisciplinary long-term follow-up for ARM patients, including a careful study of the reproductive tract to detect and treat those conditions that could affect the fertility. Moreover, an appropriate psychological support should be provided.

## Introduction

The anorectal malformations (ARM) are rare congenital anomalies affecting the distal gastrointestinal tract. They occur in one in 2500–5000 live births and encompass a spectrum of types, from the simplest cases with an expected good outcome to the rarest and most complex situations with poor functional outcome [1]. In females, the most common ARM is the rectoperineal fistula [2], followed by the rectovestibular fistula and then the cloaca. 50–80% of ARM patients have at least one other congenital malformation, affecting the genitourinary, cardiac, skeletal, and spinal apparati. The posterior sagittal anorectoplasty (PSARP) is the most common surgical technique performed to correct ARM [1]. A life-long multidisciplinary follow-up is suggested world-wide to optimize the outcome and timely manage the complications.

Female patients born with ARM are expected to have a normal life span, therefore, gynecological and psycho-sexual issues become important aspects of their adult life.

This study aimed to assess these aspects in adult females with history of ARM, to investigate the function of their genital system, the reproductive potential, and how the presence of an ARM influenced the sexual well-being.

## Materials and methods

### Population

A multifactorial evaluation of adult female patients with history of ARM was performed. Female patients aged ≥ 16 followed-up at two Italian referral centers (Treviso and Padua) were retrieved for the study. Exclusion criteria were age > 45 (perimenopause stage), previous deliveries, and severe mental retardation. Data were compared with a total of six healthy controls, chosen with the same criteria and with mean age of 26.8 years (22–30 years). All participants provided written informed consent.

### Medical, gynecological, and sexual history

The complete medical, gynecological, sexual and familiar history, including the presence of relatives affected by ARM, were collected for each patient. Moreover, the type of original malformation, comorbidities, surgical procedures and complications, bowel management, genitourinary disorders, menarche onset, and characteristics of menstrual periods were examined. Finally, some aspects of sexual activity, such as age of their first intercourse, presence of dyspareunia, vaginismus, infections, and results of previous Pap-tests, were investigated.

### Gynecologic examination, pelvic ultrasound, and cervicovaginal swab

All patients underwent a complete gynecologic examination to assess the development of secondary sexual characters, genitalia and breast. The Body Mass Index (BMI) was calculated, and secondary sexual characters were described. A pelvic ultrasound was performed to evaluate the internal genital organs. Whenever possible, speculum examination and endovaginal ultrasound were performed to obtain further information. Size and number of uterus, ovaries, endometrium, and Fallopian tubes were described. Cervicovaginal samples were collected to detect ecto- and endocervical microorganisms, such as Streptococcus, Gardnerella vaginalis, Trichomonas vaginalis, Neisseria gonorrhoeae, Chlamydia trachomatis, Mycoplasma, and Ureaplasma.

### Hormones

Each patient underwent two blood samples to dose the female sexual hormones. The first sample was taken between the 3rd and 5th day of menstrual cycle (follicular phase) to dose FSH, LH, prolactin, progesterone, 17–β–estradiol, DHEAS, testosterone, and TSH. The second sample was taken between the 20th and 22nd day of cycle (luteal phase) to dose the progesterone.

### Psycho-sexual FSFI-questionnaire

The FSFI questionnaire (Female Sexual Function Index) was administered. The FSFI is a very diffuse tool to screen the female sexual dysfunction [3], composed by 19-item self-reported tool, with six domains, scored on a scale from 0 (or 1) to 5: desire, arousal, lubrification, satisfaction, orgasm, and pain (Table 1). A total score of 26.55 is the cut-off to differentiate women with or without sexual dysfunction [4]. The validated Italian version was used in this study [5].

**Table 1:** Score system for the Female Sexual Function Index (FSFI)

Domain	Score Range	Minimum Score	Maximum Score
Desire	1–5	2	10
Arousal	0–5	0	20
Lubrication	0–5	0	20
Orgasm	0–5	0	15
Satisfaction	1–5	2	15
Pain	0–5	0	15

### Statistics

Data were expressed as mean ± standard deviation. Student’s t test was used. Statistical significance level was set at *p* < 0.05.

## Results

A total of 37 consecutive women were eligible for the study and, among these, 19 (52%) patients agreed to participate in the study, 12 (32%) could not reach the referral centers to attend the medical appointments, and 6 (16%) were not interested. The mean age of participants was 21.7 years (16–31 years). According to the Krickenbeck classification, patients were affected by cloaca (seven cases), rectovestibular fistula (six cases), imperforate anus without fistula (two cases), perineal fistula (two cases), and one each by rectovaginal fistula and non-specified low type. Eighteen patients underwent PSARP or posterior sagittal anorecto-vagino-urethroplasty (PSARVUP) during infancy; one patient born with rectovestibular fistula required surgical repair at the age of 22 years.

Ten women reported to have a good bowel management by means of diet and nine by means of trans-anal irrigation (two of them use simple enemas and seven use Peristeen^®^ bowel irrigation system). On the other hand, the most common urinary problems were recurrent urinary infections (five cases, three of them practice the intermittent catheterization).

Associated anomalies were present in 11 cases (57.8%) with a combination of affected organs: skeletal system (six cases), heart (five cases), urinary system (five cases), esophagus (two cases), filum terminale lipoma (two cases), tethered cord (one case), laryngeal schisis (one case), and Prune-Belly Syndrome (one case). Malformations regarding the genital system were present in in 36.8% (seven cases–four of them cloaca patients): three absence of uterus, two uterine septa and one each with uterus didelphys and bicornuate unicollis. Moreover, seven patients (36.8%–five of them cloaca) presented anomalies of the vagina: three stenotic and short vagina (three cloaca), and four vaginal agenesia (two cloaca and two rectovestibular fistula). Vaginal reconstruction was performed during the PSARVUP procedure in four cloaca patients, while in the other cases vaginoplasty was performed with colon (three patients), ileum (one patient), and Fortunoff flap (one patient). The gynecological examination revealed good outcome in all these patients, apart from two of them treated with PSARVUP and not yet sexually active, who showed a 2 cm vagina and an introital stenosis, respectively (Table 2).

**Table 2:** Main patients’ characteristics and gynecological findings

ID	Type of ARM	Age	Type of surgery	Genital malformations	Other malformations	Vaginoplasty	Menarche (yrs)	Menstrual periods	Intercourse	Gynecological EO	FSFI
1	Cloaca	21	PSARVUP	Short and stenotic vagina	EA type C PDA hydroureteronephrosis Thumb agenesis spondylosis	Primary repair	12	Regular with E/P therapy	Yes—Dyspareunia anorgasmia	2 cm vaginal polyp	<
2	Cloaca	26	PSARVUP	Uterus didelphys	Neurological bladder	Primary repair	12	Regular oligomenorrhea	No	Introital stenosis	<
3	Cloaca	16	PSARVUP	Bicornuate unicollis uterus short and stenotic vagina	PDA sacral anomalies	Primary repair	11	Dysmenorrhea oligomenorrhea	No	Short vagina	<
4	Cloaca	18	PSARVUP	Uterine septum	ASD tethered cord laryngeal cleft	Primary repair	13	Regular	No	Discomfort during speculum examination	<
5	Cloaca	16	PSARVUP	Short and stenotic vagina	ASD Neurological bladder	Fortunoff flap	13	Regular	No		/
6	Cloaca	16	PSARVUP	Uterus and vaginal agenesis	Lower limb absence Prune-Belly Syndrome	Colon replacement	12	Regular	No		/
7	Cloaca	16	PSARVUP	Vaginal agenesis	Single kidney VUR Sacral anomalies	Ileal replacement	14	Regular	No		/
8	Perineal Fistula	17	PSARP				12	Irregular Dysmenorrhea	No—vaginism	Pelvic floor muscle hypertonia	<
9	Perineal Fistula	20	PSARP		Epigastric hernia		14	Regular with E/P therapy	No		/
10	Rectovaginal fistula	21	PSARP				11	Dysmenorrhea oligomenorrhea	Yes—dyspareunia		<
11	Rectovestibular fistula	28	PSARP	Upper vagina and uterus agenesia	Duplex collecting system	Sigma replacement	/	/	Yes		>
12	Rectovestibular fistula	28	Neoanus reconstruction in adult life				11	Regular with E/P therapy	Yes		>
13	Rectovestibular fistula	16	PSARP				11	Dysmenorrhea	No		<
14	Rectovestibular fistula	22	PSARP		Vertebral anomalies		12	Regular oligomenorrhea	Yes		>
15	Rectovestibular fistula	17	PSARP				15	Regular	Yes		>
16	Rectovestibular fistula	31	PSARP	Vaginal and uterus agenesia		Hemirectum	/	/	Yes	Discomfort during speculum examination	<
17	No fistula	31	PSARP	Uterine septum			13	Regular with E/P therapy	Yes		>
18	No fistula	30	PSARP		EA type C Vertebral anomalies 47, XXX PDA		13	Dysmenorrhea	Yes		>
19	Unknown (low type)	21	PSARP		Tethered cord		12	Regular with E/P therapy oligomenorrhea	No		<

*EA* esophageal atresia, *PDA* patent ductus arteriosus, *ASD* atrial septal defect, *VUR* vesicoureteral reflux. FSFI: “ < ” = under the cut-off, “ > ” = over the cut-off, “/” = not filled

The mean age of menarche was 12.3 (11–15 years). Ten women reported mild dysmenorrhea and one severe, and five oligomenorrhea. At the time of the study five women were taking oral contraceptives.

**Fig. 1:**
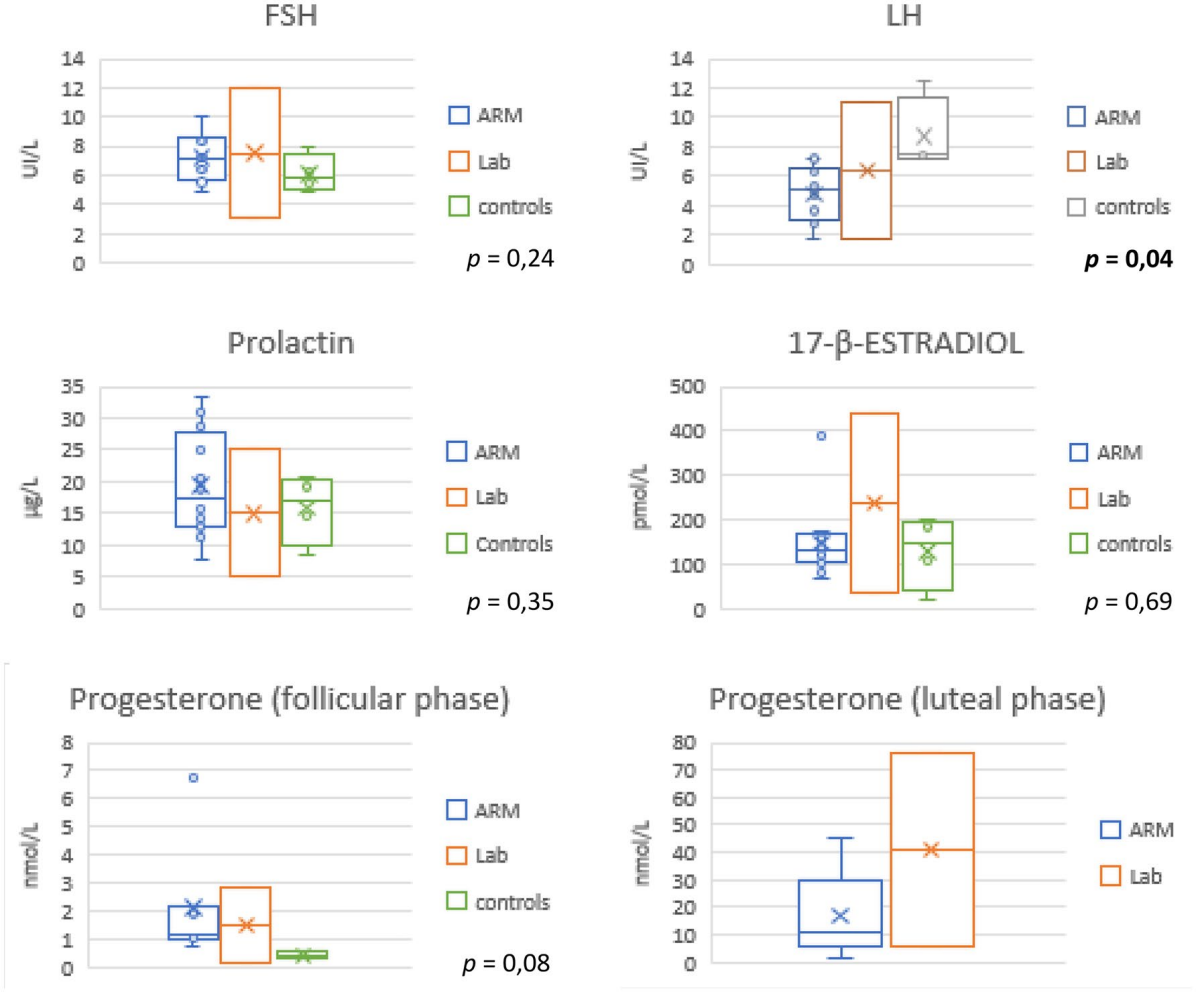
Comparison of female hormone levels in ARM cases, controls and laboratory values

Hormones’ levels were normal in our population and there was no significant difference between patients and controls (Fig. 1). Only LH levels were significantly different (4.75 UI/L vs 8.71, *p* = 0.04), but still within the normal values.

Pathogenic microorganisms were not isolated in any of our patients.

Concerning sexual aspects, nine women (47%) were sexually active but four reported at least one episode of discomfort during penetration. In particular one patient with recto-vaginal fistula described visceral pain during the intercourse and another one with cloaca was affected by dyspareunia and anorgasmia associated to a short and stenotic vagina. Three women of this group scored below the cut-off of 26.55 at FSFI questionnaire, proving an objective sexual dysfunction. Among the other 11 women (53%), four patients did not answer the FSFI questionnaire due to lack of confidence about their sexuality, the others scored under the cut-off. It is worth mentioning that a patient, born with perineal fistula, is unable to have intercourse due to severe pain during any attempt of penetration; no anatomical anomalies were detected during her gynecological examination but only vaginism and pelvic floor muscle hypertonia.

None of our patients had planned a pregnancy yet.

## Discussion

ARM patients are often complex patients who need a life-long multidisciplinary follow-up after surgical reconstruction. For this study, female patients aged ≥ 16 were enrolled to assess both their hypothalamic–pituitary–gonad axis and sexual life. The distribution of different ARM types among our patients showed an exceptional high percentage of patients born with cloaca and this explains the high incidence of associated anomalies. The patients were, indeed, enrolled from two national reference centers for ARM where more complex cases are treated.

The literature about female fertility among ARM patients is scarce because some authors describe just the gynecologic anomalies [6], some others assess their general quality of life including sexual well-being [7–9], or they report on fertility without considering the gynecologic or psychological assessment [10]. Female fertility is defined as the capacity to conceive and to produce offspring after a year of unprotected intercourse. A recent meta-analysis [10] tried to assess ARM patients’ fertility with childbirth rate as criterion, observing no statistical difference between males and females (15% vs 19%, respectively; *p* = 0.45%). On the contrary, this rate became significant in female population when less and complex ARM were compared (39% vs 14%; *p* = 0.04). This evidence suggests that, besides the correlation between severity of genital malformations and type of ARM [11], the psychosocial aspects related to the medical condition could have a higher impact in female patients. None of our patients had planned a pregnancy yet, probably because of the young age of participants (mean age 21.68 years), that is quite lower than the mean age of Italian women first pregnancy (31.9 years) [12].

Mullerian anomalies are widely represented among ARM patients (30%), in contrast to their incidence in general population (2–4%) [13]. In our sample more than half of women (52.6%) had genital abnormalities, including mild and severe, congenital and acquired. In this study, just a first-line gynecological investigation was performed but some authors suggest that also the pelvic MRI should be part of the gynecological follow-up [14]. The pregnancy is still possible in case of Mullerian anomalies, however, a worse obstetrical outcome, in terms of miscarriages and preterm labor, needs to be anticipated and cared for at specialized centers. In general, pregnancies in ARM patients must be considered as “high-risk pregnancies” and possibly referred to a center where a pediatric surgeon expert of these types of malformations is present. For most of the cases a C/section is advised to avoid overstretching and damages to the repaired pelvic floor and sphincters [15].

Despite the high percentage of associated genital malformations, patients’ sexual hormones levels were found normal and comparable to those measured in controls; same for the mean menarche age, which is consistent with the female Italian population (12.3 vs 12.4 years) [16]. That’s not unusual, in fact the hypothalamus–pituitary–ovarian axes is normally functioning in ARM patients, even in those with cloaca—who normally show more complex genital anomalies—because ovaries have a different embryological origin compared to urogenital and anorectal tracts. [17]

Vaginoplasty is normally performed in patients born with cloaca or urogenital sinus, but could be required also in rectovestibular fistula, considering that vaginal agenesia is associated in 9.5–16.3% [18]. Primary reconstruction is less invasive and more natural, but the vagina could shrink during childhood, as dilatations are not indicated and there is no hormonal stimulation at this age. On the contrary, different vaginoplasty techniques are described, the most frequently being with a tract of sigma or colon. The advantages are the possibility to achieve a good length, size, and lubrification to allow a normal sexual activity, even though the reduced fecal reservoir may favor some degree of incontinence. [19]

Sexual well-being in ARM patients is indeterminate and, often, compromised by both anatomical and psychological problems. The presence of malformed systems and related consequences, such as fecal or urinary incontinence, could affect the quality of life, self-esteem, and sexual well-being [7, 20, 21]. Recently, Eleuteri S. et al. [21] observed that the most frequent problems during sexual intercourse in female patients are loss of feces, pain and lack of vaginal lubrification. These problems are rarely discussed with the pediatric surgeons or other ARM specialists who, indeed, seem not enough confident nor competent to treat these aspects. However, the sexual well-being is considered so important that it has been recommended to address it in a multidisciplinary program [22]. About our experience, some patients referred they stopped having intercourse because of pelvic pain or vaginismus that, eventually, compromised their relationship. In some cases, a stenotic and short vaginal canal that impeded a complete gynecological examination was detected and for these women a reconstructive vaginoplasty was recommended. Three sexually active women complained dyspareunia for which a psycho-sexologist was suggested.

## Conclusions

One of the challenges in the care of ARM patients is the correct transition from the pediatric to the adult life to guarantee the same multidisciplinary follow-up they received during childhood [23]. This study confirms, indeed, the importance of a multidisciplinary long-term follow-up, that, in case of women, should include also the gynecologist, the pelvic floor trainer, and the psycho-sexologist.

## References

[1] Holschneider A, Hutson J, Peña A, Beket E (2005). Preliminary report on the International Conference for the Development of Standards for the Treatment of Anorectal Malformations. J Pediatr Surg.

[2] Jenetzky E, van Rooij I, Aminoff D (2015). The challenges of the European anorectal malformations-net registry. Eur J Pediatr Surg.

[3] Meston C (2003). Validation of the Female Sexual Function Index (FSFI) in women with female orgasmic disorder and in women with hypoactive sexual desire disorder. J Sex Marital Ther.

[4] Wiegel M, Meston C, Rosen R (2005). The Female Sexual Function Index (FSFI): Cross-validation and development of clinical cut-off scores. J Sex Marital Ther.

[5] Filocamo MT, Serati M, Li Marzi V (2014). The Female Sexual Function Index (FSFI): linguistic validation of the Italian version. J Sex Med.

[6] Vilanova-Sanchez A, Reck CA, McCracken KA (2018). Gynecologic anatomic abnormalities following anorectal malformations repair. J Pediatr Surg.

[7] Davies MC, Liao L, Wilcox DT, Woodhouse CRJ, Creighton SM (2010). Anorectal malformations: what happens in adulthood?. BJU Int.

[8] Schmidt D, Winter S, Jenetzky E, Zwink N, Schmiedeke E, Maerzheuser S (2012). Sexual function in adults with anorectal malformation: psychosocial adaptation. German Network for Congenital Uro-REctal Malformations (CURE-Net). Pediatr Surg Int.

[9] Van den Hondel D, Sloots CEJ, Bolt JM, Wijnen RMH, de Blaauw I, Ijsselstijn H (2015). Psychosexual well-being after childhood surgery for anorectal malformation or Hirschsprung’s disease. J Sex Med..

[10] Huibregtse ECP, Draaisma JMT, Hofmeester MJ, Kluivers K, van Rooij IALM, de Blaauw I (2014). The influence of anorectal malformations on fertility: a systematic review. Pediatr Surg Int.

[11] Nah SA, Ong CC, Lakshmi NK, Yap TL, Jacobsen AS, Low Y (2012). Anomalies associated with anorectal malformations according to the Krickenbeck anatomic classification. J Pediatr Surg.

[12] ISTAT. Indicatori Demografici anno 2019. Centro Diffusione Dati, Italia: 2020.

[13] Rackow BW, Arici A (2007). Reproductive performance of women with Mullerian anomalies. Curr Opin Obstet Gynecol.

[14] Fanjul M, Lancharro A, Molina E, Cerdà J (2019). Gynecological anomalies in patients with anorectal malformations. Pediatr Surg Int.

[15] Kyrklund K, Pakarinen MP, Rintala RJ (2017). Long-term bowel function, quality of life and sexual function in patients with anorectal malformations treated during the PSARP era. Semin Pediatr Surg.

[16] Rigon F, Bianchin L, Bernasconi S, Bona G, Bozzola M, Buzi F, Cicognani A, De Sanctis C, De Sanctis V, Radetti G, Tatò L, Tonini G, Perissinotto E (2010). Update on age at menarche in Italy: toward the leveling off of the secular trend. J Adolesc Health.

[17] Pradhan S, Vilanova-Sanchez A, McCracken KA, Reck CA, Halleran DR, Wood RJ, Levitt M, Hewitt GD (2018). The Mullerian Black Box: Predicting and defining Mullerian anatomy in patients with cloacal abnormalities and the need for longitudinal assessment. J Pediatr Surg.

[18] De la Torre L, Cogley K, Calisto JL, Santos K, Ruiz A, Zornoza M (2016). Vaginal agenesis and rectovestibular fistula. Experience utilizing distal ileum for the vaginal replacement in these patients, preserving the natural fecal reservoir. J Pediatr Surg..

[19] Couchman A, Creighton SM, Wood D (2015). Adolescent and adult outcomes in women following childhood vaginal reconstruction for cloacal anomaly. J Urol.

[20] Grano C, Bucci S, Aminoff D, Lucidi F, Violani C (2014). Feelings of depression in people with arm: the role of critical incidents and perceived difficulties in close and sexual relationships. Pediatr Surg Int.

[21] Eleuteri S, Aminoff D, Lucidi F, Violani C, Grano C (2019). Sexual well-being in adolescent and young adults born with arm: the perspective of the patients. Ped Surg Int.

[22] Amerstorfer G, Verhaak G-V, Miserez R-S, Schwarzer H (2019). de Blaauw, Jenetzky, van der Steeg, van Rooij,ARM-Net Consortium, What do pediatric surgeons think about sexual issues in dealing with patients with anorectal malformations?. ARM-Net Consortium members opinion Pediatr Surg Int.

[23] Giuliani S, Grano C, Aminoff D (2017). ARM-Net consortium, transition of care in patients with anorectal malformations: consensus by the ARM-Net consortium. J Pediatr Surg.

